# A Rapid and Economic In-House DNA Purification Method Using Glass Syringe Filters

**DOI:** 10.1371/journal.pone.0007750

**Published:** 2009-11-18

**Authors:** Yun-Cheol Kim, Sherie L. Morrison

**Affiliations:** Department of Microbiology, Immunology and Molecular Genetics, University of California Los Angeles, Los Angeles, California, United States of America; Institute of Evolutionary Biology (CSIC-UPF), Spain

## Abstract

**Background:**

Purity, yield, speed and cost are important considerations in plasmid purification, but it is difficult to achieve all of these at the same time. Currently, there are many protocols and kits for DNA purification, however none maximize all four considerations.

**Methodology/Principal Findings:**

We now describe a fast, efficient and economic in-house protocol for plasmid preparation using glass syringe filters. Plasmid yield and quality as determined by enzyme digestion and transfection efficiency were equivalent to the expensive commercial kits. Importantly, the time required for purification was much less than that required using a commercial kit.

**Conclusions/Significance:**

This method provides DNA yield and quality similar to that obtained with commercial kits, but is more rapid and less costly.

## Introduction

Traditional plasmid DNA purification methods all have some limitations[1]. Some are fast and allow isolation of nucleic acids within an hour[2], but speed usually comes at the price of reduced yield and/or purity[3], [4]. Although cesium chloride (CsCl) plasmid purification produces high yield and purity[3], [5], it requires extended periods (6 to 24 ours) of ultracentrifugation and the removal of CsCl and ethidium bromide is tedious and generates toxic by-products. Many commercial DNA purification kits including QIAEX II Gel Extraction Kit (Qiagen, Valencia, CA) have been developed based on the fact that DNA binds to glass milk and diatomaceous earths in the presence of chaotropic agents[6], [7]. Even though these kits are efficient, shearing forces due to fine particles may cause DNA breakage. Use of NaI (Geneclean Kit (Qbiogene, Irvine, CA)), which tends to oxidize over time, can lead to very poor DNA quality or quantity. Although glass filters have been used for small scale, high throughput plasmid purification of plasmid templates suitable for sequencing using PCR, the quantity and the quality of the plasmid purified by these methods may not be suitable for many other applications [8], [9]. Purification methods based on the fact that the large anion, DNA, can efficiently bind to positively charged resins provide high yield, however there is often contamination with genomic DNA. Although customized anion exchange resins provide efficient DNA purification, they are only available as high priced commercial kits available from vendors including Qiagen and Mackerey & Nagel.

**Figure 1:**
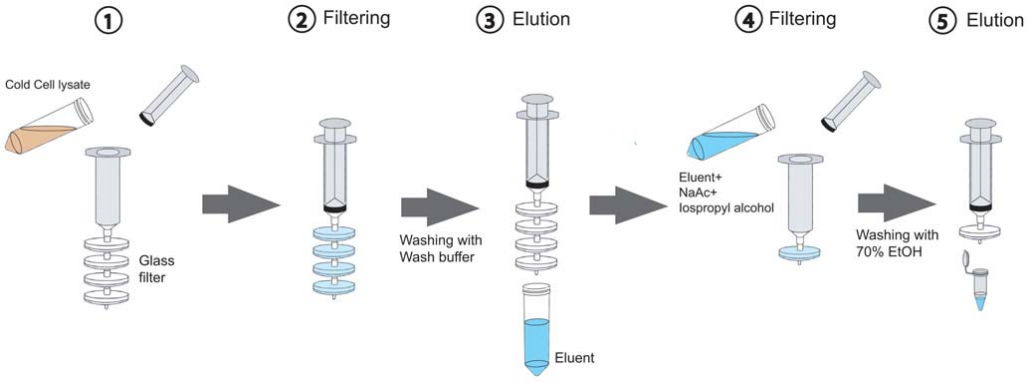
Procedure for plasmid purification using glass syringe filters. Plasmid in the cleared lysate was bound to glass syringe filters. The filters were washed with 20 ml of wash buffer and the bound plasmid was eluted with 20 ml of TE (pH 8.0). The eluent was mixed with 3 ml of sodium acetate (pH 5.1) and 23 ml of ice-cold isopropyl alcohol. The mixture was filtered through a glass filter, the filter washed with 20 ml of 70% ethanol, dried with air and bound DNA eluted with 1 ml of TE (pH 8.0).

In order to circumvent these limitations in DNA purification, we developed an efficient and economic method for DNA purification using glass syringe filters (Figure. 1). This method provides DNA yield and quality similar to that obtained with commercial kits, but is more rapid and less costly.

## Materials and Methods

### Plasmids and *E. coli* Host

pEGFP-N1 was purchased from Clonetech (Palo Alto, CA), and pLentiLoxP (pLL) 3.7[10] was obtained from Dr. Van Parijs (MIT). For pLL-LS, pLL3.7 was modified to contain an extra 4 kb of DNA. pCompact was derived from pEGFP-N1 and contained the origin of replication and kanamycin resistance gene. pCompact-GFP was made by inserting the GFP sequence into pCompact and pVSV-G was made by inserting VSV (vesicular stomatitis virus) envelope protein into pCR 3.1, purchased from Invitrogen. pGPS 2.1 and M13KE were purchased from New England Biolabs (NEB, Beverly, MA) and pCR blunt II was purchased from Invitrogen (Carlsbad, CA). pMD2-GY was made by modifying pMD2-G[11], a gift from Dr. Didier Trono (University of Geneva) to contain an extra 5 kb of DNA.

*E. coli* strains BD3.1, DH5a-F' IQ, and Top10 were purchased from Invitrogen, and BW23474 was obtained from *E. coli* Genetic Stock Center (Yale university). GM2929 was a gift from Dr. Martin Mainus (University of Massachusetts).

### Plasmid Purification

*E. coli* bearing a specific plasmid was cultured in LB for 18 hours at 37°C with shaking. The culture was centrifuged at 8,000 g for 5 minutes (Sorvall RC5C with GSA rotor) to harvest the bacteria. The cell pellet was resuspended in 10 ml of ice-cold solution I (50 mM Tris^.^HCl pH 8.0, 10 mM EDTA pH 8.0, 100 ug/ml RNase A) and lysed with 10 ml of solution II (0.2 M NaOH, 1% SDS)[12], [13]. 10 ml of solution III (3 M potassium acetate (Sigma) pH 5.3 with acetic acid) was immediately added to the lysate and the solution inverted a few times to make a protein: genomic DNA: SDS: potassium salt complex. The white precipitate was removed by centrifugation at 8,000 g for 5 minutes at 4°C (Sorvall RC5C with GSA rotor).

The volume of cleared cell lysate was measured and a half volume of ice-cold 6 M guanidine hydrochloride in 10% acetic acid was then added to make a final concentration of guanidine hydrochloride of 2 M. This cell lysate mixture was directly poured to a 50 ml syringe attached to glass syringe filters. The plunger was depressed to allow the lysate to flow through the glass syringe filter at a constant slow rate for 2 minutes (Figure. 1). Next, the filters were washed with 20 ml of wash buffer (2 mM Tris^.^HCl pH 7.5, 20 mM NaCl in 80% EtOH), and then the wash buffer was removed from the filters by repeatedly pressing air through the glass syringe filter. The bound DNA was eluted using 10 ml of TE (pH 8.0) and a constant flow for 2 minutes by pressing the plunger. The DNA in the eluent was precipitated using isopropanol/sodium acetate (2 ml of 3 M sodium acetate (pH 5.1) and 15 ml of ice-cold isopropyl alcohol). After 2 to 5 minutes incubation on ice, the mixture was added to the syringe with a new glass syringe filter, filtered as described above, and the filter washed with 10 ml of 70% ethanol. The wash buffer was removed from the filter as above, the glass syringe filter with the bound DNA attached to a new 5 ml syringe and 1 ml of TE (pH 8.0) was added. After 3 minutes incubation, the plunger was pressed repeatedly to elute the solubilized plasmid into a 1.5 ml tube. The eluted DNA was precipitated with isopropyl alcohol. The pellet was washed with 70% ethanol, and resuspended with 1 ml of TE (pH 8.0) (Figure. 1).

For the gel analysis, a 0.8% SeaKem® agarose gel (Cambrex) solution containing 0.01% ethidium bromide was made. The DNA was loaded in wells, 100 V was applied, and the DNA visualized on the transilluminator. To estimate DNA quantity, the plasmid DNA was diluted in TE (pH 8.0) so that the O.D_260_ was in the range of 0.3 to 0.6 and the absorbance measured at 260 nm and 280 nm using a spectrophotometer (BioTEK, Winooski, VT).

For side-by-side comparison, a maxi prep kit (Cat. No. 12163) from Qiagen was used to purify plasmids using the manufacture's protocol.

### Glass Syringe Filter

30 mm glass syringe filters with a 0.7 um pore-size were purchased from National Scientific Co. (F2500-18) (Quakertown, PA) and 25 mm glass syringe filters with a 1 um pore-size were purchased from Pall Life Science (4523T) (East Hills, NY). 25 mm glass filter discs with a 0.7 um pore-size, were purchased from Millipore (APFF02500) (Billerica, MA) and the filter holder was from Sterlitech (540100) (Kent, WA).

### Enzyme Digestion and Ligation

Bgl II, Afl III, Cla I, and ApaL I (NEB) were used to digest 3 ug of pLL3.7 and pLL-LS at 37°C for one hour in 60 ul using the manufacture's recommended buffer. One third of the digested DNA was stored at −20°C and the rest of the DNA was extracted with phenol: chloroform: isoamyl alcohol (25∶24∶1) and desalted with Sephadex-G25. The DNA solution was concentrated to 20 ul using a speed vacuum concentrator and ligated using 2,000 units of T4 DNA ligase (NEB) at 16°C for 18 hours. Half of the ligated DNA was extracted with phenol: chloroform: isoamyl alcohol (25∶24∶1), desalted with Sephadex-G25 and subjected to restriction enzyme digestion as described above.

### Transfection and FLOW Analysis

pEGFP-N1 was purified using a Qiagen maxi prep kit or glass syringe filters. HeLa and 293T cells were plated into 6-well plates 24 hours before transfection. Transfection with lipofectamine (Invitrogen) was carried out using the manufacture's protocol. Briefly, 3 ug of plasmid was added to 200 ul of Opti-MEM (Invitrogen) and 10 ul of lipofectamine solution was diluted in 200 ul of Opti-MEM. The two solutions were mixed, incubated for 20 minutes at room temperature, and added into the wells. The cells were fed after 24 hours and after an additional 24 hours, harvested and analyzed using a flow-cytometer (FACSCalibur (Becton-Dickinson, Franklin Lakes, NJ)).

The cells were also transfected using calcium phosphate precipitation. Briefly, 3 ug of plasmid was added to a 2.5 M calcium solution (Clonetech), mixed with 2 X HeBS (Clonetech) and after 20 minutes, the mixture was added to the cells. After 24 hours, the cells were given fresh medium and harvested after an additional 24 hours.

### DNA Purification from Agarose Gel

After restriction enzyme digestion, plasmid DNA was fractionated by agarose gel electrophoresis, the DNA band was cut from the gel, placed in a 50 ml tube, weighed and 3 volumes of gel solubilization buffer (60%(w/v) of guanidine thiocyanate (Sigma), 140 mM of MES (2-[N-Morpholino] ethanesulfonic acid) (Sigma), 0.006% phenol red (Sigma)) added. After solubilization, 1 volume (equal to the volume of the original gel fragment) of isopropyl alcohol was added. The mixture was directly added to the 50 ml syringe attached to a glass syringe filter and the solution was allowed to flow through the filter by depressing plunger for 2 minute. After washing with 20 ml of wash buffer (2 mM Tris.HCl pH 7.5, 160 mM NaCl in 80% EtOH), the filter was dried and the bound DNA eluted, phenol extracted and precipitated as described above.

## Results

In order to develop a rapid and cost effective method for plasmid purification, we have used glass filters as a DNA binding matrix because of their low price, availability and convenience (Figure.1). Glass filters are sold both as a disc and as a pre-made syringe filter. Although for convenience we primarily used the ready-made glass syringe filters, we found that filter discs placed in a holder yield the same result. Therefore, all results in this paper can be applied to both lab assembled and ready-made syringe filters. Initially, we investigated the DNA binding capacity of glass filters with 0.7 um and 1 um pore size and found that more plasmid was isolated using the 30 mm glass filter with a 0.7 um pore-size (Data not shown). Therefore all subsequent experiments were done using 30 mm glass filters with a 0.7 um pore-size.

### DNA Binding Capacity of Glass Syringe Filters

**Figure 2:**
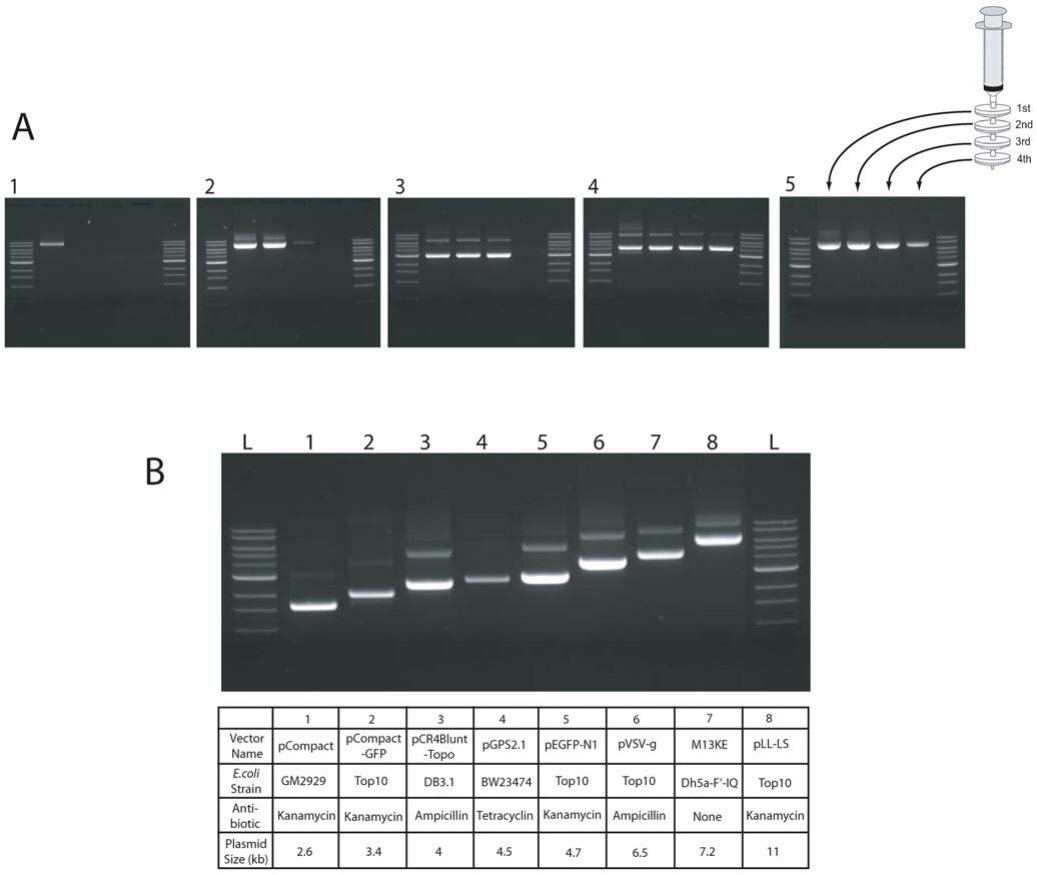
Plasmid purification from cells having different copy number plasmids with different sizes. (A) Four plasmids having a different copy number in *E. coli* were used for plasmid purification. All *E. coli* were cultured in 50 ml of LB for 18 hours at 37°C, lysates prepared as described, and plasmid purified with 4 consecutive glass syringe filters. Each plasmid bearing glass syringe filter was eluted with 1 ml TE and 5 ul was loaded on a 0.8% TAE agarose gel. 1: pMD2.GY, 2: pLL-LS, 3: pCompact-GFP, 4: pVSV.G, 5∶500 ml of *E.coli* bearing low copy number plasmid, pMD2.GY. A 1 kb ladder marker (NEB) is shown in the outside lanes of each gel. (B) *E.coli* bearing different plasmids were cultured in 50 ml LB and the plasmid purified from cell lysates using 4 glass syringe filters. The isolated plasmids were resuspended in 1 ml TE (pH 8.0) and 2 ul was loaded on to a 0.8% TAE agarose gel. L: 1 kb ladder (NEB).

To test the binding capacity of the glass syringe filters, we used 4 glass syringe filters in series and plasmids having different copy number. The plasmid copy number is as follows: pMD2GY < pLL-LS < pCompact-GFP < pVSV-G. We found that each glass syringe filter captures plasmid DNA until it reaches its maximum binding capacity after which unbound plasmid DNA goes to the next filter where it is bound (Figure. 2(A)). When a low copy number plasmid was used (pMD2-GY), the first filter captured all available plasmid (Figure. 2(A), lane 1). With a high copy number plasmid, all 4 glass syringe filters were saturated with plasmid (Figure. 2(A), lane 4). Using a series of filters makes it possible to recover all plasmid efficiently from the cell lysate.

To determine the capacity of a single glass syringe filter, cell lysate from 50 ml of *E.coli* culture bearing pEGFP-N1 (the lysate contained about 300 ug of plasmid) was applied to a single glass syringe filter and eluted with 1 ml of TE (pH 8.0). The total DNA was measured by spectrophotometer and the single filter was found to capture up to 150 ug of plasmid DNA (data not shown).

When a plasmid having low copy number (pMD2-GY) is used, the relative concentration of plasmid DNA in the cell lysate is low (Figure. 2(A), panel 1). Increasing the culture volume of *E.coli* bearing pMD2-GY to 500 ml increased the plasmid yield (Figure. 2(A), panel 5). The purified plasmid had a 260/280 nm ratio of 1.88 indicating that the capacity and specificity of DNA binding to glass filters was not compromised by a low concentration of plasmid in the presence of large amounts of impurities.

### Glass Syringe Filter Purification of Plasmids of Different Size

In order to determine whether the binding of DNA to glass filters depends on plasmid size, plasmids ranging from 2.6 to 11 kb in size were purified from a 50 ml *E.coli* culture, eluted with 1 ml of TE (pH 8.0) and analyzed by gel electrophoresis (Figure. 2(B)). Purification did not depend on plasmid size, the strain of *E.coli* or the antibiotic selection. The glass syringe filter method was also used successfully to purify the RF form of M13 phage (Figure. 2(B), lane 7).

### Quality of the Plasmid Purified by Glass Syringe Filter

**Figure 3:**
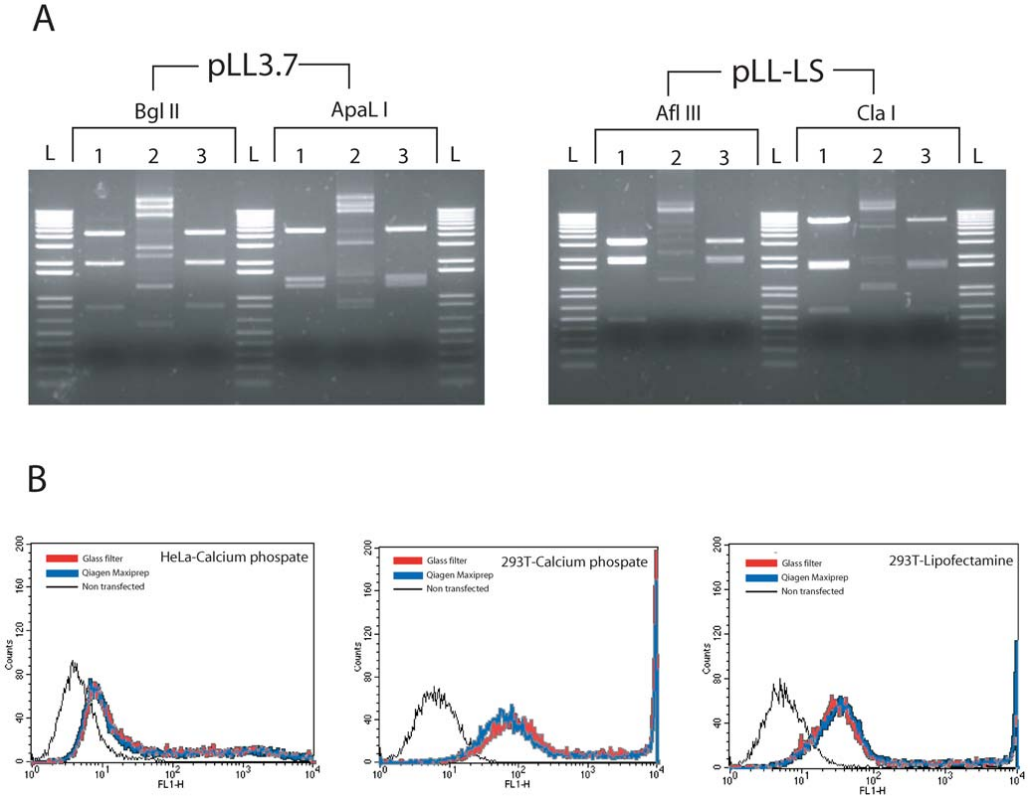
Test of plasmid quality using restriction enzyme digestion, ligation and transfection. (A) Plasmids purified by glass syringe filters were digested with the indicated restriction enzymes, the digested plasmid was ligated and then re-cut with the same restriction enzyme. 1: Initial restriction enzyme digestion. 2: Ligation at 16°C for 12 hours. 3: Re-digestion with same enzyme. L: 1 kb ladder (Invitrogen) (B) GFP expression following transfection with purified pEGFP-N1. *E.coli* bearing pEGFP-N were cultured in 200 ml LB and plasmid purified using either a Qiagen Maxi-prep kit or glass syringe filters. The plasmid was quantitated by spectrophotometer and transfected into 293T and HeLa cells using lipofectamine or calcium phosphate. After 2 days, the transfected cells were harvested and GFP expression was analyzed by FLOW.

Purified plasmids are frequently used for restriction enzyme digestion and ligation. Therefore, plasmids purified by glass syringe filter were digested with different restriction enzymes, two-thirds of the digested DNA was ligated and then the half of the ligation mixture was re-digested with the same restriction enzymes. Purified DNA was cut with the three restriction enzymes used and all digested DNA was also efficiently ligated. When the ligated DNA was re-cut by same restriction enzyme, the same DNA pattern was observed as seen with the first digestion (Figure. 3(A)). Therefore, the quality of DNA purified by glass syringe filter was suitable for restriction enzyme digestion, the digested DNA was efficiently ligated, and the ligated DNA could be re-digested indicating that the ends of the digested DNA remained intact.

The quality of DNA is known to affect the transfection efficiency of mammalian cells. Therefore, we compared the transfection efficiency of plasmids purified using either glass syringe filters or Qiagen Maxi-prep kits (Figure. 3(B)). pEGFP-N1 purified by both methods gave rise to the same level of GFP expression when HeLa cells were transfected using calcium phosphate precipitation. Also, there were no differences in level of GPF expression when both plasmid preparations were transfected into 293T cells using either calcium phosphate precipitation or lipofectamine[3]. The experiments were repeated three times with the same results. Therefore, plasmid purified using glass syringe filters appears to be of the same quality as plasmid DNA purified using a Qiagen Maxi-prep kit.

### Comparison of Methods for DNA Isolation

**Figure 4:**
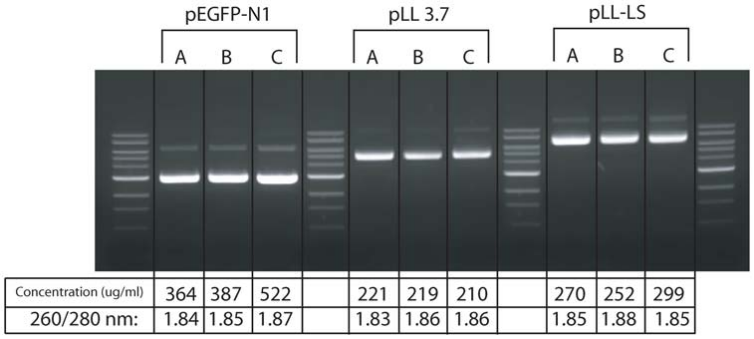
Comparison of plasmid yield using different purification and precipitation methods. Bacterial cultures of each plasmid were divided into three equal portions. The first was purified using the Qiagen Maxi prep kit, and the plasmid eluted from the column was precipitated by isopropyl alcohol after overnight incubation at −20°C using centrifugation (Lane A). The second portion was also purified using the Qiagen Maxi prep kit but the plasmid eluted from the column was isolated by glass syringe filter filtration (Lane B). For the last part of the culture, the plasmid was purified by 4 glass syringe filters and harvested by glass syringe filtration. (Lane C). The final volume of all plasmid preparations was 1 ml in TE (pH 8.0) and 5 ul was loaded on to a 0.8% TAE agarose gel.

In order to further compare the quantity and quality of plasmid DNA purified by glass syringe filter with that of DNA purified using the Qiagen Maxiprep kit, we purified three plasmids (pEGFP-N1, pLL3.7 and pLL3-LS) using both methods. A 150 ml culture of *E.coli* bearing each plasmid was divided into three 50 ml parts. Two were purified using a Qiagen Maxi-prep kit and the one was purified by the glass syringe filter method (Figure. 4). Current procedures for efficient DNA precipitation are time consuming requiring extended incubation at cold temperature and/or extended centrifugation time at high speed. Therefore we developed a new rapid method for efficiently concentrating plasmid DNA from large volumes of a DNA: alcohol mixture. Even when commercial kits are used, DNA must be precipitated with isopropyl alcohol from large volumes of eluent.

We found that the fine DNA precipitates in the alcohol: DNA mixture are bound to glass filters. To compare the yield of plasmid precipitated by centrifugation following an overnight incubation at cold temperature to the yield obtained following glass syringe filter capture, both eluents (15 ml each) from the Qiagen-Maxi kits were precipitated with isopropyl alcohol. One was incubated at −20°C overnight and then harvested by centrifugation at 3700 g for 30 minutes (Figure. 4, a). The other was immediately filtered through a glass syringe filter and eluted with TE (pH 8.0) after washing with 70% ethanol as described above. Plasmid from the third aliquot was prepared using the glass syringe filter method (Figure. 4, c) as described above. All three plasmids gave the same patterns on agarose gel with similar yield. The 260/280 nm ratios were in the 1.8 to 1.9 range indicating that the DNA was pure. Therefore, harvesting DNA precipitate using glass syringe filter was as efficient as DNA precipitation following overnight incubation in the cold. The yield using the glass syringe filter capturing method was greater than that obtained using centrifugation without an overnight cold temperature incubation (Data not shown). A single glass syringe filter can bind up to 900 ug of plasmid DNA from the alcohol: DNA mixture (data not shown). Therefore, this step can reduce the time required for plasmid purification without decreasing the yield. Furthermore, plasmid purification using the glass syringe filter yielded DNA of the same quantity and quality as did the Qiagen maxi-prep kit.

### DNA Purification from Agarose Gel

**Figure 5:**
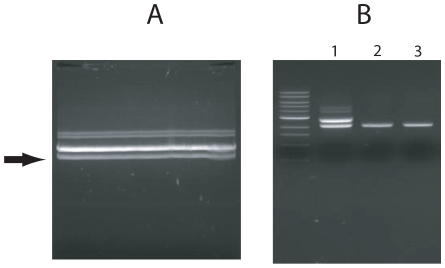
DNA purification from agarose gel. Digested plasmid was fractionated in an agarose gel (A) and a specific band (arrow) cut from the gel. The gel was divided in two and DNA from half was purified by four Qiagen gel extraction columns (2 in B) and the DNA from the other half was purified by a single glass syringe filter (3 in B). The original digestion mixture before purification is included (1 in B). The purified bands were analyzed on a 0.8% TAE agarose gel.

Glass filters can bind DNA in the presence of a high concentration of a chaotropic agent and this characteristic can be used to purify DNA from agarose gels solubilized with guanidine isothiocyanate, a chaotropic agent. We explored several conditions and found that 60% (w/v) guanidine isothiocyanate in 140 mM of MES (2-[N-Morpholino] ethanesulfonic acid) buffer was the optimal gel-melting buffer for the purification of DNA from agarose gels. DNA was separated by agarose gel electrophoresis, one band isolated and the agarose fragments containing the DNA divided in half. One half was purified using four Qiagen gel extraction columns and the other half was purified using a single glass syringe filter as described above. The quantity of DNA purified using the glass syringe filter was equivalent to that purified by the four Qiagen gel extraction columns (Figure. 5). This purification method provides a particularly good alternative if a large quantity of DNA needs to be purified.

## Discussion

Purification of plasmid DNA from *E.coli* can be tedious and time consuming, but is critical for many experimental procedures. Although it can be simple and easy to use commercial kits for plasmid purification, most kits are expensive. The most widely used kits, including Qiagen-Maxi-prep kits, utilize anion exchange resins to capture plasmid DNA from crude bacterial lysates. Because the flow rate of the cell lysate through the column depends on gravity, it can take 1 to 1.5 hours for the whole procedure.

To circumvent these problems, we have developed a rapid and economic method to purify plasmid DNA that provides an alternative to the expensive and time-consuming protocols of commercial kits. The cost per 1 ug of plasmid DNA prepared using glass syringe filters is less than a third of that for DNA prepared using commercial kits (Table. 1). This method can be completed within 20 to 30 minutes without sacrificing either plasmid purity or yield. The method is versatile because the number of glass syringe filters used can be varied based on culture volume and plasmid copy number. As a rule of thumb, 200 ml of an overnight *E.coli* culture bearing a high or medium copy number plasmid yields about 500 ug of DNA that can be bound using four glass syringe filters. About 500 ml of *E.coli* culture was required for plasmids with low copy number for a similar yield.

**Table 1:** Comparison^(1)^ of the three plasmid purification methods.

Methods	Qiagen Maxi Prep	Glass syringe filter (4 filters)	Glass filter disc (4 discs)
Time ^(2)^	1.5 to 2 hours	20 to 30 minutes	20 to 30 minutes
Total cost	$16.36 ^(3)^	$6.84 ^(4)^	$4.34
Restriction enzyme digestion	Good	Good	Not tested
Transfection	Good	Good	Not tested
Yield	300 to 500 µg	400 to 600 µg	400 to 600 µg
Cost/µg DNA	3.3 to 5.5 cents	1.1 to 1.7 cents	0.7 to 1.1 cents

(1)This comparison was obtained based on retail price for each reagent because commercial kit for this method is not currently available.

(2)The time required for each method is the period necessary to get purified plasmid DNA starting with a 200 ml *E.coli* culture. For Qiagen Maxiprep kit (Cat. No.: 12163) [14], time was calculated from the manufacture's instructions.

(3)The cost of Qiagen Maxiprep kit (Cat. No.12163) is $ 409 per 25 preparations so that the cost of each preparation is $16.36. This cost calculation doesn't include any additional laboratory materials that are required.

(4)The cost includes 5 glass syringe filters ($ 1 each), a 60 ml syringe ($ 1) and the required reagents.

There are several important considerations in obtaining optimal plasmid yield. A critical step in plasmid purification is the use of the appropriate neutralization solution. Although guanidine hydrochloride with 99% purity is recommended for making the neutralization solution, less expensive guanidine hydrochloride of lower purity (98%) can be used if insoluble materials are removed by centrifugation and filtration through a 0.45 um filter. Appropriate removal of cellular debris from the cell lysate by centrifugation is also important for efficient binding of plasmid to the glass filter. For optimal yield, it is important that the cleared cell lysate has a final concentration of guanidine hydrochloride of 2 M. The temperature of the cleared lysate also influences the efficiency of plasmid capture by glass syringe filters with a cold lysate giving a 10% higher yield than a room temperature lysate (data not shown).

Because the maximum capacity of a glass syringe filter for soluble plasmid DNA in the presence of chaotropic agents is about 150 ug, 4 glass syringe filters can purify up to 600 ug of plasmid from a cleared cell lysate. In contrast, the capacity of a glass syringe filter for harvesting DNA from an alcohol: DNA mixture was up to 900 ug because in this case, there is nonspecific capturing of fine DNA precipitates on the glass syringe filter. We found the optimal condition for producing the precipitate was the addition of an equal volume of ice-cold isopropyl alcohol and 0.15 volumes of 3 M sodium acetate (pH 5.1) followed by a 2 to 5 minute incubation on ice. Although it was not feasible to precisely control the flow rate through the glass filter, we found empirically that a slower filtration rate resulted in greater plasmid yield. We have found that this method works well when a few plasmids are purified at the same time. However, a special multi-channel vacuum apparatus would probably be required if this method were adapted to high-throughput plasmid purification.

Although the purification of DNA from agarose gels is routine, it is difficult and costly to purify large amounts of DNA using commercial gel extraction kits because of the limited capacity of the available columns. We have shown that a single glass syringe filter can be used to purify up to 100 ug of DNA from an agarose gel and that this can be easily scaled up for larger amounts of DNA by attaching several glass syringe filters in tandem. The gel-melting buffer described was optimized both for pH and concentration of guanidine isothiocyanate (data not shown).

In summary, we describe a simple, cost effective, and rapid method for purifying plasmid DNA and harvesting DNA from agarose gels. This method will provide an alternative method for efficient and flexible plasmid purification.
